# Transcriptional Analysis of *Spodoptera frugiperda* Sf9 Cells Infected with *Daphnis nerii* Cypovirus-23

**DOI:** 10.3390/ijms26157487

**Published:** 2025-08-02

**Authors:** Wendong Kuang, Jian Yang, Jinchang Wang, Chenghua Yan, Junhui Chen, Xinsheng Liu, Chunhua Yang, Zhigao Zhan, Limei Guan, Jianghuai Li, Tao Deng, Feiying Yang, Guangqiang Ma, Liang Jin

**Affiliations:** 1 Institute of Microbiology, Jiangxi Academy of Sciences, Nanchang 330029, China; jemappelleyangjian@zju.edu.cn (J.Y.); wangjinchang75@163.com (J.W.); allenchen0426@gmail.com (J.C.); zgzhan_iom@163.com (Z.Z.); glmnh@126.com (L.G.); jeremy_leakey@sina.com (J.L.); dengtao95@outlook.com (T.D.); 2 Institute of Biological Resources, Jiangxi Academy of Sciences, Nanchang 330029, China; ellenyung@foxmail.com (C.Y.); jxaasyfy@163.com (F.Y.); 3 School of Life Sciences, Jiangxi University of Traditional Chinese Medicine, Nanchang 330004, China; 4 State Key Laboratory of Virology, Wuhan Institute of Virology, Chinese Academy of Sciences (CAS), Wuhan 430071, China; yanchenghua23@126.com (C.Y.); maguangqiang@163.com (G.M.); 5 State Key Laboratory for Animal Disease Control and Prevention, Lanzhou Veterinary Research Institute, Chinese Academy of Agricultural Sciences, Lanzhou 730050, China; liuxinsheng@caas.cn

**Keywords:** *Daphnis nerii* cypovirus-23, Sf9 cells, transcriptome analysis

## Abstract

*Daphnis nerii* cypovirus-23 (DnCPV-23) is a new type of cypovirus that has a lethal effect on many species of Sphingidae pests. DnCPV-23 can replicate in *Spodoptera frugiperda* Sf9 cells, but the replication characteristics of the virus in this cell line are still unclear. To determine the replication characteristics of DnCPV-23 in Sf9 cells, uninfected Sf9 cells and Sf9 cells at 24 and 72 h after DnCPV-23 infection were collected for transcriptome analysis. Compared to uninfected Sf9 cells, a total of 188 and 595 differentially expressed genes (DEGs) were identified in Sf9 cells collected at 24 hpi and 72 h, respectively. KEGG analyses revealed that 139 common DEGs in two treatment groups were related to nutrition and energy metabolism-related processes, cell membrane integrity and function-related pathways, detoxification-related pathways, growth and development-related pathways, and so on. We speculated that these cellular processes might be manipulated by viruses to promote replication. This study provides an important basis for further in-depth research on the mechanism of interaction between viruses and hosts. It provides additional basic information for the future exploitation of DnCPV-23 as a biological insecticide.

## 1. Introduction

*Daphnis nerii* cypovirus-23 (DnCPV-23) was initially isolated from naturally diseased larvae of *Daphnis nerii* (*D. nerii*), which belongs to the order Lepidoptera and family Sphingidae, and is a worldwide pest [1] that mainly damages the leaves of the ornamental plant oleander and the medicinal plants *Rauvolfia vomitoria* Afzel and *Catharanthus* [1,2]. Compared with other cypoviruses, this new type of cypovirus has different electrophoretic migration patterns and conserved terminal sequences [1,3,4]. In addition to *D. nerii*, DnCPV-23 can infect and induce death in various harmful insects of the Sphingidae family. However, as a potential biopesticide, the molecular mechanism of the interaction between DnCPV-23 and its hosts remains unclear. The study of the interaction mechanism between DnCPV-23 and hosts is essential for understanding virus virulence and virus genome function, screening host molecules that promote virus replication, and screening complex drugs that improve virus insecticidal efficacy.

We previously performed a transcriptome analysis of DnCPV-23-infected and uninfected *D. nerii* midguts [1]. DnCPV-23 can infect wild insects such as *D. nerii*, but the lifecycle of these insects is strongly affected by season and cannot be artificially reproduced at present. Therefore, it is essential to study the effects of DnCPV-23 on cell lines. In addition, DnCPV-23 infection of the *D. nerii* midgut occurs in the form of occlusion-derived viruses (ODVs) in vivo, and the midgut cell composition is complex. However, the Sf9 cell line can be infected in vitro with free virus particles of DnCPV-23. Based on different infection forms and cell types, we speculate that the transcriptome differs between cultured cell lines and *D. nerii* midguts. Our previous results revealed that DnCPV-23 can be effectively replicated and passaged in Sf9 cells [5]. However, the molecular mechanism underlying DnCPV-23 infection in Sf9 cells has not been fully elucidated. Moreover, there are few reports on the replication of insect RNA viruses in heterologous cell lines. *Perina nuda* virus (PnV) can establish persistent infection in a heterologous *Lymantria xylina* cell line, NTU-LY. Various methods were used to investigate virus replication in NTU-LY cells [6]. Moreover, the honeybee virus deformed wing virus (DWV) can infect the heterologous *Lepidopteran haemocytic* cell line P1 cells [7]. This study reports the replication of another RNA virus, DnCPV-23, in the heterologous cell line Sf9. Our work provides an important foundation for in-depth research on the replication of RNA viruses in heterologous cells and for establishing a sustained infection cell model for insect RNA viruses. To investigate how Sf9 cells react to DnCPV-23 infection, we used a high-throughput sequencing approach to assess the impact of DnCPV-23 infection on global gene expression in Sf9 cells and analysed the host factors that may affect virus replication. In this study, uninfected Sf9 cells and DnCPV-23-infected Sf9 cells were collected at 24 h and 72 h after infection for transcriptome analysis to investigate changes in the gene expression profiles of Sf9 cells after virus infection and identify the host signalling pathways that may affect virus replication.

We speculate that upregulating some processes (including nutrition and energy metabolism, cell membrane integrity and functions, detoxification, and growth and development) after viral infection is beneficial for DnCPV-23 replication. For example, juvenile hormone acid O-methyltransferase-like (JHAMT) can promote virus proliferation by prolonging insect pupation time; virus replication can be promoted by inhibiting host apoptosis through upregulating the expression of cytochrome P450 9e2 (*CYP9E2*); and Sequestosome 1 (p62/SQSTM1) affects the lifecycle of viruses by regulating autophagy. Through this study, we have identified some host genes that may affect the replication of DnCPV-23. In future work, we will further verify the interaction between these genes and DnCPV-23, which provides an important theoretical basis for understanding the interaction between viruses and hosts, virus replication in heterologous cell lines, and viral biological insecticide development.

## 2. Results

### 2.1. Viral Infection of Cells

**Figure 1:**
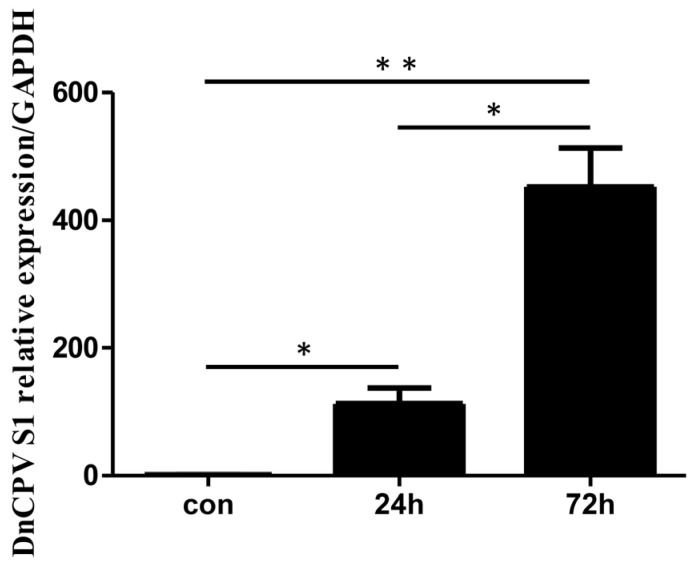
Analysis of viral RNA in infected cells. Sf9 cells were collected at 24 and 72 h post-infection (hpi). The mRNA levels of DnCPV-23 *S1* in control cells and infected cells were assessed via qRT–PCR. Statistically significant differences are indicated as * *p* < 0.05, ** *p* < 0.01. Data with *p* values < 0.05 were considered statistically significant.

The virus-to-cell ratio is 0.2 polyhedra per cell. Prior to transcriptome analysis, qRT–PCR was used to assess the mRNA levels of the DnCPV-23 *S1* gene in infected cells (samples collected at 24 h and 72 h post-infection (hpi)) and in uninfected cells. The results revealed higher relative expression of viral gene mRNA in infected cells than in uninfected cells (Figure 1).

### 2.2. Overview of Transcriptome Sequencing

All of the samples were sequenced independently. Transcriptome sequencing analysis of 15 samples resulted in a total of 81.39 Gb of clean data. The clean data of each sample ranged from 6.41 to 7.04 G. The Q30 of each sample ranged from 94.12% to 94.42%, and the average GC content was 45.96%. The genome alignment of each sample was obtained by mapping reads to the reference genome, with a mapping rate of 81.42% to 82.46%. A total of 595 DEGs were identified, with 506 being upregulated and 89 being downregulated between the CPV_72h samples and the control samples.

### 2.3. Effects of Viral Infection on the Transcriptome Expression of Sf9 Cells

**Figure 2:**
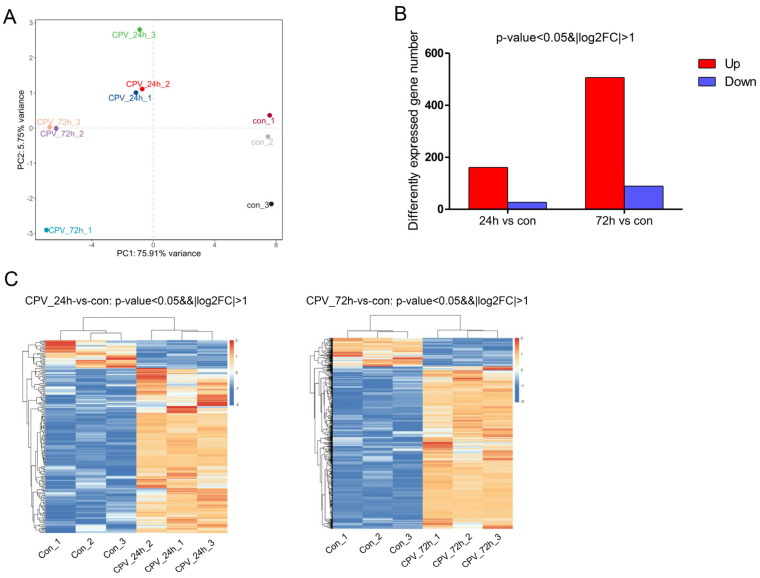
Influence of DnCPV-23 infection on the Sf9 cell transcriptome. (**A**). Plot of the 1st and 2nd principal components of sample variation, as determined using principal component analysis. (**B**). Compared with uninfected cells, in infected cells, 161 and 506 genes were upregulated (red bars), and 27 and 89 genes were downregulated (blue bars) at 24 hpi and 72 hpi, respectively. (**C**). Heatmap of 188 and 595 differentially expressed genes (DEGs) between uninfected cells and cells collected at 24 hpi and 72 hpi.

As shown in Figure 2, PCA1 accounted for 75.91%, and PCA2 accounted for 5.75%. Therefore, the percentage of the total of the two was 81.66%, thus accounting for a high proportion and representing the overall population to a large extent. The principal component analysis revealed a clear separation of the samples among the three groups (Figure 2A), indicating that the samples had good repeatability. Compared with uninfected cells, in infected cells, the number of upregulated genes at 24 hpi and 72 hpi was 161 and 506, respectively, and the number of downregulated genes at 24 hpi and 72 hpi was 27 and 89, respectively (Figure 2B). In addition, a heatmap of the gene expression data is presented in Figure 2C. The results suggested that these DEGs could be used to distinguish the samples. The results revealed that viral infection could influence Sf9 gene expression at different time points.

### 2.4. Analysis of DEGs

**Figure 3:**
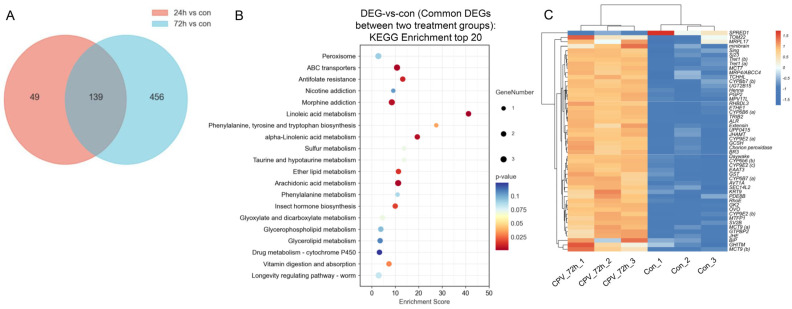
Signalling pathways and genes involved in the interaction between DnCPV-23 and Sf9 cells. (**A**) Venn diagram of DEGs obtained from the two treatment groups; there were 139 DEGs that exhibited differential expression between samples infected with DnCPV-23 for 24 h and 72 h compared to the control. (**B**) KEGG classifications of 139 common DEGs between the two treatment groups (top 20). The larger the bubble is, the greater the number of DEGs. The bubble colour changes (purple–blue–green–red) indicate that the smaller the enrichment *p* value is, the greater the significance. (**C**) Heatmap analysis for 50 DEGs with the highest expression level selected from the overlapping 139 DEGs.

We analysed 139 DEGs shared by the two treatment groups for further analysis (Figure 3A). KEGG functional enrichment analysis was performed using the common DEGs between the two treatment groups to identify the relevant biological pathways associated with these DEGs. Based on the enrichment score obtained from the KEGG analysis, we identified the 20 most enriched pathways. These pathways play important roles in nutrition and energy metabolism-related processes (for example, “linoleic acid metabolism” and “vitamin digestion and absorption”), cell membrane integrity and function-related pathways (for example, “glycerophospholipid metabolism” and “ether lipid metabolism”), detoxification-related pathways (for example, “ABC transporters”), growth and development-related pathways (for example, “insect hormone biosynthesis”), and insect immune responses (for example, “arachidonic acid metabolism”) (Figure 3B). We selected 50 DEGs with the highest expression levels from these 139 DEGs for heatmap analysis. A heatmap of the gene expression data is presented in Figure 3C. The results suggested that the identified DEGs could distinguish the samples and that DnCPV-23 infection influenced gene expression in Sf9 cells.

Figure 3C shows the top 50 genes with the highest expression levels among the overlapping 139 DEGs, and the relevant information of these genes is presented in Table 1.

### 2.5. qRT–PCR Validation of DEGs

**Figure 4:**
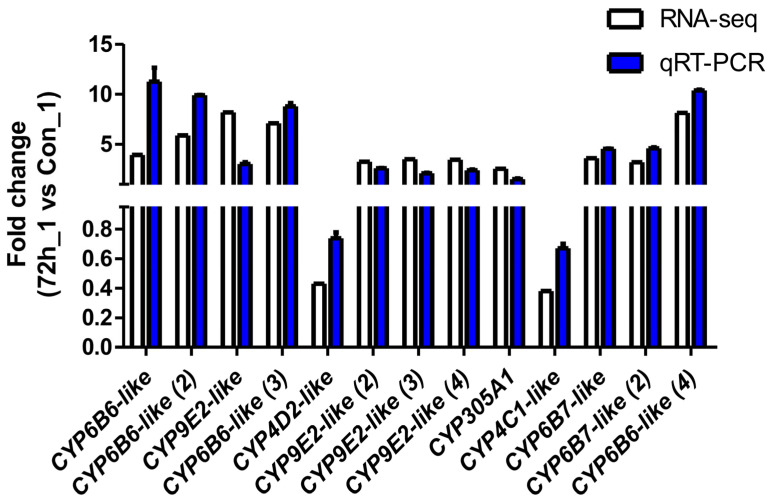
Validation of RNA-seq profiles by real-time qPCR. To validate the RNA-seq data, the relative mRNA levels of 13 selected DEGs in infected cells (CPV_72_1) were examined by qPCR. The mRNA levels determined via qPCR are presented as the fold change compared with the mRNA levels in the uninfected cells (Con_1) after normalisation against *GAPDH*. The relative expression levels from the RNA-seq analysis were calculated as RPKM values. The error bars represent means ± SEMs.

To verify the reliability of the transcriptome data and the DEG results obtained by RNA-seq, thirteen DEGs were selected for qPCR analysis. As shown in Figure 4, the fold-change values for the infected cells (CPV_72_1) vs. the uninfected cells (Con_1) obtained via the qPCR analysis were consistent with the values obtained via RNA-seq for all of the selected genes.

## 3. Discussion

To analyse the important signalling pathways and genes that may be involved in the interaction between DnCPV-23 and Sf9 cells, we selected 139 common genes between 24 h DEG and 72 h DEG for analysis. The KEGG analysis revealed that many processes changed in Sf9 cells after virus infection, including nutrition and energy metabolism-related processes (such as “linoleic acid metabolism” and “vitamin digestion and absorption”), cell membrane integrity and function-related pathways (such as “glycerophospholipid metabolism” and “ether lipid metabolism”), detoxification-related pathways (such as “ABC transporters”), and growth and development-related pathways (such as “insect hormone biosynthesis”). The KEGG analysis indicated that these processes or pathways might have important roles in the interactions between DnCPV-23 and Sf9 cells. Furthermore, these processes or pathways also changed in the midgut of *Daphnis nerii* after DnCPV-23 infection [1].

DnCPV-23 infection can affect the expression of critical genes involved in energy metabolism, such as glycerol kinase-like (*GK*; Gene ID: LOC118276660) and trehalose transporter 1 (*Tret1*; Gene ID: LOC118271571, LOC118279019). Glycerol kinase (GK) is an enzyme that catalyses the formation of glycerol 3-phosphate from ATP and glycerol [8]. *GK2* is a glycerol biosynthesis gene that plays a crucial role in survival during the cold period [9]. HCV/HBV infection reduces the expression of miR-451a, which inhibits *GK* expression, and miR-451a attenuates hepatitis C virus replication by targeting glycerol kinase. Furthermore, the supplementation of miR-451a could impede lipid deposition, reduce steatohepatitis, and inhibit HCV replication in the liver [10]. After virus infection, two *Tret1* transcripts were significantly upregulated. Tret1 is a specific and high-capacity facilitated transporter of trehalose, which is the major sugar found in insect haemolymph fluid, providing energy and promoting growth, metamorphosis, stress recovery, chitin synthesis, and virus replication in insects [11,12,13]. In BmN cells, BmNPV infection promotes the expression of trehalose hydrolysis and transport-related genes to facilitate BmNPV proliferation through the trehalose-PI3K-Akt pathway in the midgut [14]. Moreover, trehalose could facilitate virus replication and shedding of dengue virus (DENV) in *Aedes aegypti* cells. It is possible that trehalose increases DENV2 infection in Aag2 cells by promoting autophagy to prolong cell survival and enhance virus maturation, and enhancing virus cell entry through the modification of cell membranes [12].

In addition to affecting energy metabolism, DnCPV-23 infection also affected insect metamorphosis, day–night rhythm, and oocyte development-related genes. Juvenile hormone esterase-like (*JHE*, Gene ID: LOC118263046) was upregulated, induced by DnCPV-23 infection. JHE is the primary juvenile hormone (JH)-specific degradation enzyme that plays a crucial role in regulating JH levels. Depletion of JHE resulted in the extension of *Bombyx mori* larval stages [15] or larval mortality in *Telchin licus* (Lepidoptera) [16]. JHE activity is downregulated in larval *Adoxophyes honmai* following dhoNPV and AdorNPV infection, and small interfering RNAs were proposed to play a role in the downregulation of *JHE* gene expression in baculovirus-infected caterpillars [17]. The *Mamestra brassicae* Multiple Nucleopolyhedroviruses (MbMNPV) infection of *H. armigera* larvae inhibits the expression of JHE following the upregulation of JH titre. The upregulation of JH titre prevents the pupation of *H. armigera* and promotes MbMNPV replication through the JH-Met-Kr-h1 signalling pathway [18]. DnCPV-23 infection promoted the expression of juvenile hormone acid O-methyltransferase-like (*JHAMT*; Gene ID: LOC118265567). Juvenile hormone (JH) titres in insects are regulated by synthesis with JHAMT and catabolism with JHE [19]. JH could regulate metamorphosis, reproduction, diapause, and polyphenisms [20,21]. The knockdown of *JHAMT* reduced JH titre, leading to larval death in *Aedes aegypti* [20] and changing the phenotype of *S. frugiperda* [22]. During the early stages of infection, Zika virus (ZIKV) regulates the expression of some ribosomal protein genes (*RpL23* and *RpL27*) through the JH-Met-Tai signalling pathway. Moreover, the upregulated expression of *RpL23* and *RpL27* could facilitate the translation of viral proteins and promotes ZIKV infection in *Aedes aegypti* [23]. Further research is needed to investigate the detailed effects of these host genes on DnCPV-23 infection.

After viral infection, the expressions of many detoxification-related genes were upregulated, such as cytochrome P450 9e2 (*CYP9E2*), cytochrome P450 6B7 (*CYP6B7*), cytochrome P450 6B6 (*CYP6B6*), glutathione S-transferase (*GST*), UDP-glucuronosyltransferase (*UGT*), and multidrug resistance-associated protein 4-like (*MRP4/ABCC4*). *CYP9E2* (Gene ID: LOC118264059, LOC118264054, LOC118262727), *CYP6B7* (Gene ID: LOC118279736, LOC118279835) and *CYP6B6* (Gene ID: LOC118263048, LOC118262642) are involved in xenobiotic detoxification in insects. In *Helicoverpa armigera*, *CYP6B6* plays crucial roles in the transformation of esfenvalerate and capsaicinoids [24,25]. In *H. armigera*, *CYP6B7* is known as an important detoxification gene in response to fenvalerate [26,27]. In honeybees, thiacloprid can be specifically metabolised by CYP9E2 [28]. In addition, the *CYP9E2* gene of *Bombyx mori* can be regulated by the microRNA bmo-miR-31-5p to inhibit apoptosis and promote BmNPV proliferation [29]. In the future, we will further analyse the role of these *CYP450* genes in virus replication. *GST* (Gene ID: LOC118261931) is a group of enzymes associated with detoxification and plays an important role in detoxifying endogenous and exogenous compounds [30]. The expression of *GST* could be regulated by viral infection [31,32]. GST may modify lipids to inhibit viral infection, or regulate inflammatory-like mediators to exert antiviral effects [33]. However, GST could promote BmNPV proliferation through regulating glutathione (GSH) levels [34]. DnCPV-23 infection also induced the expression of another detoxification enzyme, *UGT2B15-like* (Gene ID: LOC118278849). UDP-glucuronosyltransferases (UGTs) could transform various exogenous and endogenous compounds, which play a critical role in detoxification and homeostasis in insects [35]. Moreover, UGTs could regulate viral infection. It is speculated that UGT33D1 reduces oxidative stress to promote BmNPV infection [36]. DnCPV-23 replication could promote the expression of *MRP4/ABCC4* (Gene ID: LOC118262225). *MRP4* (multidrug resistance-associated protein 4) is a member of the ATP-binding cassette (ABC) transporter C subfamily, which are transporters of various substrates including endogenous compounds, xenobiotic compounds, and nutrients [37,38]. HIV-1 infection induced a significant increase in *MRP4* expression in human macrophages. The increase in *MRP4* expression may favour the efflux of antiretroviral drugs in macrophages to promote virus replication [39].

DnCPV-23 could upregulate the expression of *Sequestosome 1* (*p62/SQSTM1*, Gene ID: LOC118273096, LOC118273082). BmCPVs can induce mitophagy, which is a form of autophagy, through the interaction of VP4 with host Tom40 to promote self-replication [40]. Additionally, BmCPV replication could be attenuated by vsp21-induced autophagy [41]. Thus, the impact of autophagy on virus replication may be opposite in different situations. Many studies have shown that autophagy is a double-edged sword for virus replication [42,43,44]. We used autophagy inhibitors to analyse the effects of blocking different stages of the autophagy process on virus replication. Compared with the control (DMSO), treatment with Baf A1 significantly restricted virus replication, whereas treatment with 3-MA or CQ did not affect virus replication (Supplementary Figure S1). The results suggested that maintaining the acidic pH and protein degradation ability of lysosomes might be crucial for virus replication [45,46]. In addition, we found that DnCPV-23 infection promoted the expression of *Sequestosome 1* (*SQSTM1/p62*), an adaptor protein of selective autophagy, to promote the degradation of aggregate-prone proteins. SQSTM1 is a well-known autophagy cargo receptor (ACR) that plays a dual role during pathogen infection; it can promote the degradation of viral proteins, aiding in the elimination of the virus, but can also be manipulated by viruses to promote mitochondrial degradation, thereby suppressing immune responses [47]. A previous study reported that Baf A1 treatment can decrease the protein level of SQSTM1 [48]. Above all, we speculate that the DnCPV-23 infection of host cells promotes autophagy and self-replication by upregulating *SQSTM1* expression, which can be blocked by Baf A1. However, in this study, we did not explore it thoroughly. To confirm this conclusion, further research is needed to analyse the impact of lysosomal pH on DnCPV-23 replication and elucidate whether Baf A1 affects the replication of DnCPV-23 by downregulating the expression of *SQSTM1*. In the future, we will (1) use CRISPR/Cas9 to knock out *SQSTM1* in Sf9 to determine whether Baf A1 inhibits DnCPV replication through SQSTM1; (2) use immunofluorescence, electron microscopy scanning, Western Blotting, and RT qPCR to determine which stage of the virus Baf A1 specifically affects: (3) use knockdown, overexpression, or inhibitor inhibition assays to analyse the host factors involved in Baf A1 affecting virus replication [49,50,51].

In Sf9 cells, we revealed substantial differences in the transcription of genes related to energy metabolism, growth, development, and detoxification processes induced by DnCPV-23 replication. We speculate that these cellular processes might be regulated by DnCPV-23 to promote its replication. In addition, we found that autophagy-related genes also undergo changes, and we need further research to determine the role of autophagy in the DnCPV-23 replication. In these pathways, we analysed some specific host genes that may be involved in DnCPV-23 replication. This study provides important preliminary work for future validation experiments. This study analysed the differentially expressed genes (DEGs) of Sf9 cells after DnCPV-23 infection for the first time, and identified host genes that may be involved in DnCPV-23 replication. Our work enriched the research on the interaction between cypovirus and hosts. However, there has been no definitive and credible validation of the role of relevant host genes in virus replication, making it difficult for readers to determine which host genes are truly involved in the lifecycle of DnCPV-23. In summary, the results obtained in this study provide an important foundation for further in-depth research on the mechanism of interaction between DnCPV-23 and its hosts.

## 4. Materials and Methods

### 4.1. Cell Line, Virus Stock, Inhibitors, and Antibodies

Sf9 cells, an ovary-derived cell line of *Spodoptera frugiperda*, were cultured at 28 °C in Grace’s insect medium (Gibco, Waltham, MA, USA) supplemented with 10% foetal bovine serum (Gibco, USA). DnCPV-23 was initially isolated from the larvae of *D. nerii* and propagated in *D. nerii* larvae [1]. The suspension of DnCPV-23 polyhedra used for infecting Sf9 cells was stored at 4 °C in the dark. 3-Methyladenine (3-MA) (Selleck, Houston, TX, USA) was used at 5 mM, chloroquine (CQ) (MedChemExpress, Monmouth Junction, NJ, USA) was used at 40 µM, and bafilomycin A1 (Baf A1) (MedChemExpress) was used at 20 nM. For immunoblotting, rabbit polyclonal antibodies against the DnCPV-23 protein encoded by the viral S1 gene (ABclonal Biotechnology, Woburn, MA, USA) were used at a 1:500 dilution, and mouse monoclonal antibodies against tubulin (Abbkine, Atlanta, GA, USA) were used at a 1:10,000 dilution.

### 4.2. Virus Inoculation

To promote virus infection in cells, purified DnCPV-23 polyhedra at a concentration of approximately 2 × 10^7^/mL were treated with 0.2 M Na_2_CO_3_–NaHCO_3_ (pH 10.8), after which the pH was adjusted to 7.4 with 1 M Tris-HCl (pH 6.8) buffer. The free virus particles were stored at −80 °C. Sf9 cells were seeded in 6-well plates, and when they reached a confluency of approximately 75%, they were infected with free DnCPV-23 virus particles (50 μL of the viral suspension containing free DnCPV-23 particles without polyhedral particles). After incubation for 12 h, the cell culture medium was replaced with Grace medium containing 10% (*v*/*v*) FBS, and the cells were harvested 24 h and 72 h after infection. Uninfected cells were used as controls.

### 4.3. RNA Extraction, Library Preparation, and RNA-Seq

Control Sf9 cells and Sf9 cells infected with DnCPV-23 were collected at 24 h and 72 h postinfection. All RNA sequencing (RNA-seq) procedures were conducted by the Oebiotech Company (Shanghai, China). Total RNA was extracted from Sf9 cells using TRIzol reagent (Invitrogen, Waltham, MA, USA) in accordance with the manufacturer’s protocols. The RNA integrity and concentrations were assessed using an Agilent 2100 Bioanalyzer (Agilent Technologies, Santa Clara, CA, USA). Nine RNA samples (including three uninfected samples and nine infected samples) with confirmed RNA integrity were used to construct the libraries. The cDNA libraries were prepared using a TruSeq RNA Sample Preparation Kit (Illumina, San Diego, CA, USA) in accordance with the manufacturer’s protocols. Thereafter, the obtained cDNA libraries were sequenced on the Illumina HiSeq2500 platform, which generated paired-end raw reads of 150 bp.

### 4.4. RNA-Seq Data Analysis

To obtain clean reads, raw reads in fastq format were first processed using fastp 1, and the low-quality reads were removed. The clean reads were mapped to the reference genome ZJU_Sfru_1.0 GCF_011064685.1 using HISAT2 (version 2.2.4) [52]. Gene expression levels were normalised as fragments per kilobase of transcript per million mapped reads (FPKM) values. Principal component analysis (PCA) was performed using R (v 3.2.0) to evaluate the biological duplication of samples.

Differentially expressed genes (DEGs) were analysed using DESeq2. A Q value < 0.05 and a fold change > 2 or fold change < 0.5 were set as the thresholds for DEGs. Hierarchical cluster analysis of DEGs was performed using R (v 3.2.0) to investigate the expression patterns of genes in different groups and samples. Furthermore, all DEGs were subjected to GO and KEGG analyses to screen for significantly enriched terms via R (v 3.2.0).

### 4.5. Quantitative Real-Time PCR

Quantitative real-time PCR (qRT–PCR) was used to analyse the expression level of the DnCPV-23 *S1* gene in transcriptome samples and verify the DEGs identified by RNA-seq. Total RNA was isolated from samples for transcriptomic analysis using TRIzol reagent (Life Technologies, Carlsbad, CA, USA) and then treated with DNase I (Fermentas, Glen Burnie, MD, USA). Total RNA (500 ng per sample) was reverse-transcribed into complementary DNA (cDNA) using a PrimeScript RT Reagent Kit (Takara, San Jose, CA, USA). Then, qRT–PCR was performed using Talent qPCR PreMix SYBR Green (Tiangen, Beijing, China) on a QuantStudio™ 7 Flex Real-Time PCR System (Applied Biosystems™, Waltham, MA, USA). One cycle was added for melting curve analysis for all of the reactions to verify the product specificity. Relative expression levels of genes were calculated using the 2^−ΔΔCT^ method, and GAPDH was used as a reference for normalisation [53]. All of the primers for the target genes are listed in Table 2.

### 4.6. Cell Treatments

Sf9 cells were seeded (1 × 10^5^ cells per well) in a 24-well plate and allowed to attach overnight. The next day, the cells were pretreated with the autophagy inhibitors 3-MA, CQ, and Baf A1 for 2 h prior to DnCPV-23 infection. The final concentrations of 3-MA, CQ, and Baf A1 were 5 mM, 40 µM, and 20 nM, respectively. Two hours later, 10 μL of the viral suspension containing free DnCPV-23 particles without polyhedral particles was added to the cells for 6 h. After the incubation period, the medium was aspirated, and fresh cell culture media containing autophagy inhibitors were added to the cells, which were subsequently cultured for 24 h and 48 h.

### 4.7. Western Blotting

Cells were collected by centrifugation at 300× *g* for 5 min at 4 °C, and total protein was extracted using RIPA lysis buffer (Beyotime, Shanghai, China). The cells were placed on ice for 20 min, and the supernatant was obtained by centrifugation at 6000× *g* for 6 min at 4 °C. Samples were mixed with 5× SDS loading buffer and boiled at 100 °C for 7 min. For Western blot analysis, after SDS-PAGE, proteins were transferred to polyvinylidene fluoride (PVDF) membranes, which were incubated overnight at 4 °C with the appropriate primary antibodies. The membranes were subsequently rinsed three times with TBST (each for 10 min) and incubated with horseradish peroxidase-conjugated goat anti-mouse IgG or goat anti-rabbit IgG as the secondary antibody for 2 h at room temperature. Protein bands were visualised using an enhanced chemiluminescence (ECL) Western blot detection kit and analysed using the ChemiScope 3000 mini system (Clinx, Shanghai, China).

### 4.8. Statistical Analysis

Statistical analysis was performed using GraphPad Prism 6.0. The values are presented as the mean plus or minus the standard error of the mean (SEM). The unpaired two-tailed *t*-test was used to compare one factor among different groups. Statistically significant differences are indicated as * *p* < 0.05, ** *p* < 0.01, or *** *p* < 0.001. Data with *p* values < 0.05 were considered statistically significant.

## Figures and Tables

**Table 1:** Information of 50 DEGs with the highest expression levels selected from the 139 common DEGs in two treatment groups (P62/SQSTM1 only differentially expressed at 72 hpi).

Gene_ID	Gene Name	Abbreviation of Gene Name	Average Expression of CPV_72h	Fold Change of CPV_72h vs. Con	Impact of Gene on Virus Replication
LOC118281152	GTP-binding protein 2-like	*GTPBP2*	79.60941971	2.939862906	Unknown
LOC118281850	tribbles homolog 2-like	*T* *RIB* *2*	83.6385479	3.116434931	Unknown
LOC118276660	glycerol kinase-like	*G* *K* *2*	27.26451044	2.891990222	Related to HCV replication
LOC118263046	juvenile hormone esterase-like	*JHE*	44.06177965	2.213259729	Inhibits MbMNPV replication
LOC118278849	UDP-glucuronosyltransferase 2B15-like	*UGT2B15*	52.08919252	3.208530207	May promote virus replication
LOC118272911	FAD-linked sulfhydryl oxidase ALR-like	*ALR*	72.24821537	3.384447759	Unknown
LOC118264059	cytochrome P450 9e2-like	*CYP9* *E* *2 (a)*	65.3765198	3.155621533	Promotes BmNPV replication
LOC118273268	growth hormone-inducible transmembrane protein-like	*G* *HITM*	39.35624854	2.069179782	Unknown
LOC118275035	mitochondrial fission process protein 1-like	*MTFP1*	39.59756816	3.266534569	Unknown
LOC118268094	UPF0415 protein C7orf25 homolog	*UPF0415*	30.07996836	2.787196551	Unknown
LOC118264054	cytochrome P450 9e2-like	*CYP9* *E* *2 (b)*	25.4264185	2.914285755	Promotes BmNPV replication
LOC118276320	phosphoglycolate phosphatase 2-like	*PGP2*	46.20605215	4.101708059	Unknown
LOC118274261	synaptic vesicle glycoprotein 2B-like	*SV2B*	15.33200925	2.282949456	Unknown
LOC118267668	persulfide dioxygenase ETHE1, mitochondrial-like	*ETHE1*	27.24655442	3.715194818	Unknown
LOC118276246	chorion peroxidase-like	*C* *horion peroxidase*	8.672305521	3.575164367	Unknown
LOC118271617	rho-related GTP-binding protein RhoE-like	*R* *ho* *E*	8.074911214	3.360901616	Unknown
LOC118263839	balbiani ring protein 3-like	*BR3*	12.93191276	2.824491018	Unknown
LOC118276116	circadian clock-controlled protein daywake-like	*D* *aywake*	26.71343267	5.011601593	Unknown
LOC118264074	rhomboid-related protein 3-like	*RHBDL3*	6.692910582	3.302604069	Unknown
LOC118268821	protein henna-like	*H* *enna*	8.512926579	2.48340151	Unknown
LOC118275579	monocarboxylate transporter 9-like	*MCT9 (a)*	7.291586508	2.356887011	Unknown
LOC118267228	protein singles bar-like	*S* *ing*	14.17416077	3.521595539	Unknown
LOC118279835	cytochrome P450 6B7-like	*CYP6* *B* *7 (a)*	6.115680087	2.885802312	Unknown
LOC118262727	cytochrome P450 9e2-like	*CYP9* *E* *2 (c)*	7.514131089	7.340344336	Unknown
LOC118268856	glycine cleavage system H protein-like	*GCSH*	17.70414895	4.804584058	Unknown
LOC118262982	endoplasmic reticulum chaperone BiP	*BiP*	4.831579472	2.149337031	Unknown
LOC118264312	amino acid transporter AVT1A-like	*AVT1A*	6.275652722	4.412106553	Unknown
LOC118263447	transcriptional regulator ovo-like	*OVO*	2.995905576	2.958866706	Unknown
LOC118279736	cytochrome P450 6B7-like	*CYP6* *B* *7 (b)*	5.224798912	3.233611488	Unknown
LOC118263048	cytochrome P450 6B6-like	*CYP6* *B* *6 (a)*	7.812977386	6.348864958	Unknown
LOC118268820	extensin-like	*E* *xtensin*	4.414752748	4.159429339	Unknown
LOC118269313	mitochondrial import receptor subunit TOM22 homolog	*TOM22*	7.870868019	2.250525698	Unknown
LOC118271571	facilitated trehalose transporter Tret1-like	*Tret1 (a)*	4.258888116	2.285612846	Promotes BmNPV replication
LOC118267488	serine/threonine-protein kinase minibrain-like	*M* *inibrain*	8.060249965	2.327461242	Unknown
LOC118279019	facilitated trehalose transporter Tret1-like	*Tret1 (b)*	4.17484285	2.427275019	Promotes BmNPV replication
LOC118274614	monocarboxylate transporter 7-like	*MCT7*	5.706508283	2.048856356	Unknown
LOC118261931	glutathione S-transferase 1-like	*GST*	14.72725987	5.251990067	Promotes BmNPV replication
LOC118276669	sprouty-related, EVH1 domain-containing protein 1-like	*SPRED1*	1.72739488	0.41233409	Unknown
LOC118266697	SEC14-like protein 2	*SEC14L2*	3.912579123	3.121802027	Unknown
LOC118267308	mpv17-like protein	*MPV17L*	8.924007294	2.829362881	Unknown
LOC118276314	monocarboxylate transporter 9-like	*MCT9 (b)*	3.561204881	2.266039059	Unknown
LOC118265759	high affinity cAMP-specific and IBMX-insensitive 3′,5′-cyclic phosphodiesterase 8-like	*P* *DE* *8* *B*	0.91473512	2.468483098	Unknown
LOC118276616	23 kDa integral membrane protein-like	*Sj23*	2.825832687	2.633377326	Unknown
LOC118262642	cytochrome P450 6B6-like	*CYP6* *B* *6 (b)*	4.842834846	5.279997561	Unknown
LOC118267867	39S ribosomal protein L17, mitochondrial-like	*MRPL17*	9.329088079	2.393602125	Unknown
LOC118265812	excitatory amino acid transporter 3-like	*EAAT3*	2.680965274	4.855375004	Unknown
LOC118265567	juvenile hormone acid O-methyltransferase-like	*JHAMT*	2.885832192	4.064307053	May promote ZIKV infection
LOC118262225	multidrug resistance-associated protein 4-like	*MRP4/ABCC4*	1.162929203	3.585355174	Promotes HIV-1 replication
LOC118280766	keratin, type I cytoskeletal 9-like	*KRT9*	2.440427412	2.243261847	Unknown
LOC118270457	trichohyalin-like	*TCHHL*	2.221009547	3.830439946	Unknown
LOC118273096	sequestosome-1-like	*p62/SQSTM1*	97.47736978	2.15174043	Plays a dual role in viral infection
LOC118273082	sequestosome-1-like	*p62/SQSTM1*	82.74454193	2.035871822	Plays a dual role in viral infection

**Table 2:** Primers used for the qRT–PCR analysis of viral RNA in transcriptome samples and for the validation of RNA-seq data.

No.	Primer Name	Primer Sequence (5′ to 3′)	Tm (°C)	Gene ID	Target Gene
1	S1-RTPCR-F	GTGCTGATGGTCTGCTAA	49.6	N/A	DnCPV-23 *S1*
2	S1-RTPCR-R	TGATTGATGACGACATTGAG	51.5		
3	1893F	GTATTAATCCTACTGTACCACTACG	51.7	LOC118261893	*CYP6B6-like*
4	1893R	CTCTTCTTTGCTACGAGATTAGG	52.4		
5	2642F	TACTAGAGGTGAGGTGAGTGATAT	53.4	LOC118262642	*CYP6B6-like*
6	2642R	CAGCGTGTAGTTCTTTAGGATATG	52.7		
7	2727F	TTCGAGACAGTATCATCAGGAATG	53.7	LOC118262727	*CYP9E2-like*
8	2727R	GACACAACCATATCCATATAGACC	52.2		
9	3048F	CTCAAGCATTCTTCTTCTTCTTAGC	53.3	LOC118263048	*CYP6B6-like*
10	3048R	GTCTACTTCATCCTGTACCTTCTT	53.2		
11	3400F	GGTACTTCAGTAGTGGTGAATATC	52	LOC118263400	*CYP4D2-like*
12	3400R	GAGATAGTGATCTTGAGTTCCATC	52.1		
13	4054F	GTCTACCAGTGTTCACCTTTATTAG	52.5	LOC118264054	*CYP9E2-like*
14	4054R	CATTAGTGACCTTCGCTATGAGAT	53.8		
15	4059F	GATCAACATCCTCATGGAAGTTAAG	53	LOC118264059	*CYP9E2-like*
16	4059R	CGATATGTGACTCTTCAACAGTTG	53.2		
17	4410F	CAAGATTGTCAGGAACGATATGATC	53	LOC118264410	*CYP9E2-like*
18	4410R	CGATATGTGACTCTTCAACAGTTG	53.2		
19	6520F	TAGACGCAGTAATAGGAGATAGAC	52.4	LOC118266520	*CYP305A1-like*
20	6520R	CTATAGGGTAGTATCTATGGACTTC	50.5		
21	0774F	GAGAGTAATTATCGTCGGTTGAAG	52.6	LOC118270774	*CYP4C1-like*
22	0774R	CATAGTCCATAGTCGGGTAGTATT	52.7		
23	9736F	GAGGTACTCTCAGTCTCTAAGTA	51.7	LOC118279736	*CYP6B7-like*
24	9736R	CGTTATACGAGGCTATACATAGG	51.6		
25	9835F	AGTGAGACCTCAAGAGATAATGAC	53.2	LOC118279835	*CYP6B7-like*
26	9835R	CTTGTAGTCTCTCTTCCTCAGTAT	52.5		
27	2155F	CTGTAGCGTTATATGATCTCTTCC	52.1	LOC118282155	*CYP6B6-like*
28	2155R	GTCCAATAGGCTTGTAGTTTCTCT	53.9		
29	gapdhF	GTGCCAAGAAGGTCATCAT	52.2	N/A	*GAPDH*
30	gapdhR	GAGAGGAGCGAGACAGTT	53.8		

## Data Availability

The original contributions presented in this study are included in the article/Supplementary Material. The original data of the transcriptome will be released on 10 July 2025 or upon publication. BioProject accession: PRJNA1279750.

## Supplementary Materials

The following supporting information can be downloaded at https://www.mdpi.com/article/10.3390/ijms26157487/s1.
