# Supplementary figures and images for: Transcriptional Analysis of Spodoptera frugiperda Sf9 Cells Infected with Daphnis nerii Cypovirus-23
Source: Int J Mol Sci. 2025 Aug 2;26(15):7487. doi: 10.3390/ijms26157487 (PMC12347268; doi:10.3390/ijms26157487)

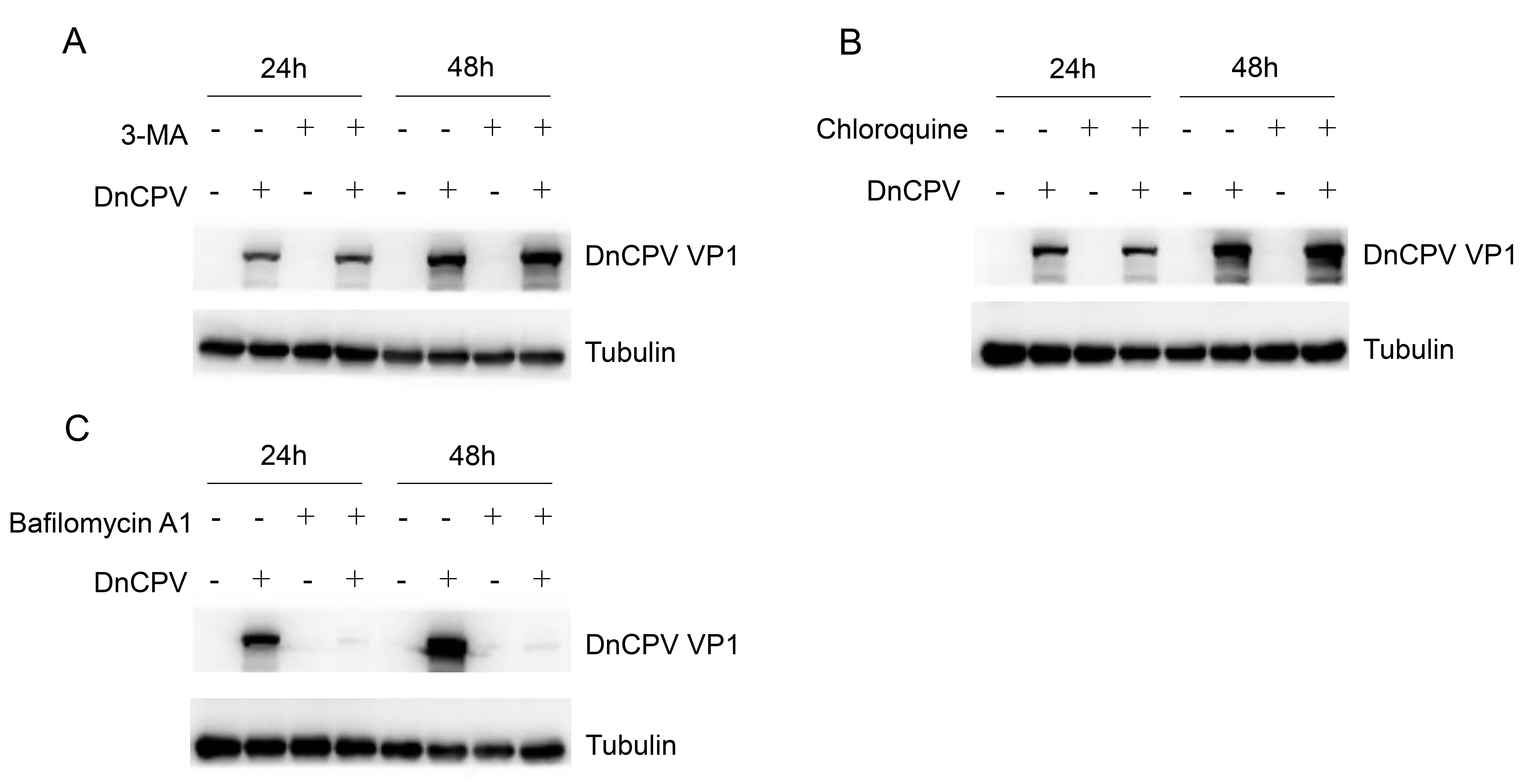

Supplement: Supplementary file 1 [file ijms-26-07487-s001.zip › sfig 1.png]
